# The Diagnostic Value of CSF α-Synuclein in the Differential Diagnosis of Dementia with Lewy Bodies vs. Normal Subjects and Patients with Alzheimer’s Disease

**DOI:** 10.1371/journal.pone.0081654

**Published:** 2013-11-25

**Authors:** Elisabeth Kapaki, George P. Paraskevas, Evangelia Emmanouilidou, Kostas Vekrellis

**Affiliations:** 1 National and Kapodistrian University of Athens, School of Medicine, 1st Department of Neurology, Eginition Hospital, Athens, Greece; 2 Division of Basic Neurosciences, Biomedical Research Foundation of the Academy of Athens, Athens, Greece; UCL Institute of Neurology, United Kingdom

## Abstract

The detection of α-synuclein (α-syn) in the cerebrospinal fluid (CSF) of patients with synucleinopathy has yielded promising but inconclusive results. The aim of the present study was to determine the diagnostic value of α-syn as a biological marker for Dementia with Lewy bodies (DLB) vs. normal subjects and patients with Alzheimer’s disease (AD), after strict control of several recognized confounders. Sixteen patients with DLB, 18 patients with AD and 22 age- and sex-matched normal controls (CTRL) were recruited. The levels of total α-syn in CSF were measured using a novel enzyme-linked immunosorbent assay. There was a significant increase of CSF α-syn levels in DLB patients as compared to the CTRL and AD groups (P= 0.049 and 0.01 respectively). ROC analysis revealed that increased α-syn was 81.8% specific for the discrimination of DLB vs. CTRL and 90% vs. AD. However, sensitivity was lower (56.2 % and 50% respectively). These findings provide evidence for a possible diagnostic role of α-syn as a surrogate biomarker for DLB.

## Introduction

Early diagnosis of dementing neurodegenerative disorders such as Alzheimer’s disease (AD) and Dementia with Lewy Bodies (DLB) is considered necessary for a number of reasons, including treatment initiation at the earliest stage [1]. Cerebrospinal fluid (CSF) biomarkers may offer a good tool for early diagnosis, since CSF directly interacts with the extracellular space of the brain, giving a clue of pathophysiological processes [2]. In AD, CSF biomarkers have been recognized to reflect the prevailing hypothesis for its pathogenesis and the typical biochemical profile is a decrease of Aβ42 levels, which is considered to reflect amyloidogenesis, as well as an increase of total tau (T-tau) and phosphorylated tau (P-tau), which reflect axonal degeneration and tangle formation [3,4]. The above mentioned biomarkers have been incorporated in the recent diagnostic criteria established by the National Institute on Aging and the Alzheimer’s Association (NIA-AA), recommending their use in order to increase the diagnostic confidence in establishing the presence (or the absence) of AD pathophysiological processes [5].

DLB is considered to be the second most common type of neurodegenerative dementia in the elderly, characterized by fluctuating cognition, parkinsonism and psychiatric symptoms. Although the current clinical diagnostic criteria appear very specific, they are much less sensitive due to substantial clinical as well as pathological overlap mainly with AD and other dementia or parkinsonian syndromes. Patients with DLB typically do not exhibit tau pathology, but they often have Aβ pathology. The core protein involved in its pathogenesis is α-synuclein (α-Syn), which is the major component of Lewy bodies, also present in Parkinson’s disease (PD) [6]. It has been shown that α-Syn is present in detectable amounts in CSF of normal subjects and PD patients [7], with its origin to be mostly brain derived [8] and thus it has received much attention as a possible biomarker in synucleinopathies [9]. However, studies measuring CSF α-Syn levels in DLB have yielded conflicting results showing either decreased levels [10-15], or no difference between patients and controls [16-21].

The discrepancy among these studies probably results from several factors, including variation in antibodies that might detect different species of α-Syn, limited numbers of patients and differences in the control group (normal subjects vs. “other neurological diseases”). In addition, there was inadequate control for important confounding factors, such as diurnal variation, rostro-caudal gradient within CSF, gender- or age-dependence and, importantly, blood contamination of CSF.

The aim of the present study was to determine the diagnostic value of CSF α-Syn levels to discriminate DLB from healthy controls as well as AD (the most common dementia interfering with its differential diagnosis), after strict implementation of the current proposed recommendations on standardized operating procedures (SOPs) for the CSF biomarkers [22]. For this purpose, we have used a recently developed, in-house ELISA, for the accurate quantification of full length α-Syn.

## Materials and Methods

### 1: Patients

The study was performed according to the ethical guidelines of the 1964 Declaration of Helsinki. It was conducted in two stages and had the approval of the local committee of Eginition hospital. Both patients and controls gave written informed consent to participate in the study. Both patients and controls gave standard (written) informed consent to participate in the study. In cases that this was not feasible, the next of kin carer takers or guardians consented on the behalf of participants whose capacity to consent was compromised.

**Figure 1:**
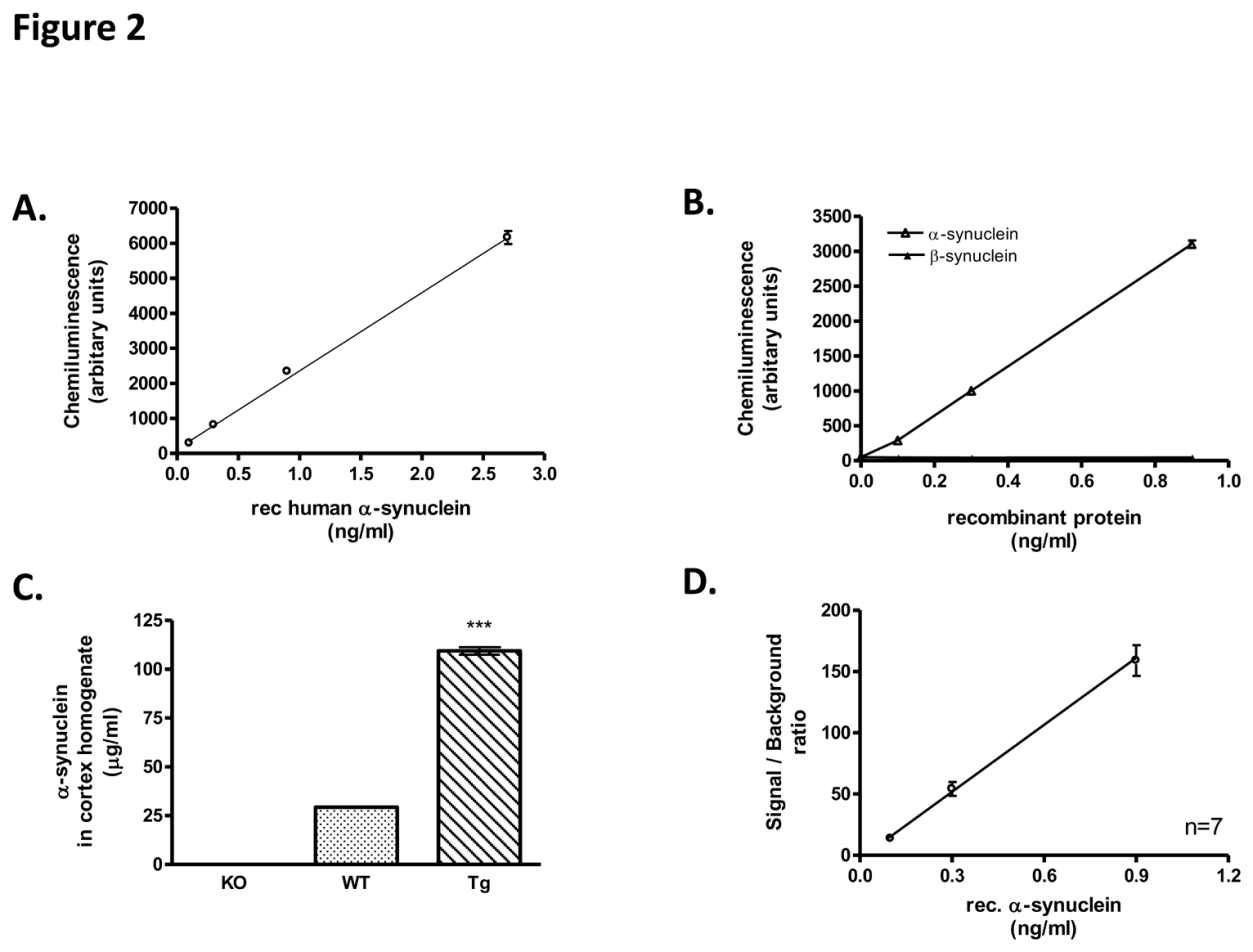
Analytical characteristics of α-Syn ELISA. (A) Generation of a standard curve of the α-Syn ELISA in the low concentration range (0.1-2.7 ng/ml) showing the assay sensitivity (5 pg/ml). (B) Assessment of assay specificity by measuring different concentrations of recombinant α-Syn and β-Syn (0.1-0.9 ng/ml). (C) ELISA specificity was tested by measuring brain homogenates from KO, WT and α-Syn Tg mice. No α-Syn was detected in the KO animals. α-Syn levels in Tg brain extracts were 3.7-fold higher than in WT (***: p<0.001, n=4, 1-way ANOVA test followed by Tukey’s test) (D) Calibration graphs for the assessment of the day-to-day reproducibility of the assay during a period of 6 months (n=7 curves). The average of the S/B ratios for each concentration were plotted vs. the input of recombinant α-Syn. Error bars correspond to the reproducibility of the assay.

During the 1st stage, a total of 19 control CSF samples from healthy subjects were used (CTRL batch 1). They came from our CSF Bank and had been collected during 2006. They comprised of otherwise healthy, elderly individuals that had undergone minor surgery (such as hernia repair or knee joint surgery) under spinal anesthesia. They suffered no neurological, psychiatric or other major disease and their cognitive function was within normal limits as detected by history, semi-structured interview and MiniMental State Examination (MMSE) [23] prior to the operation. Routine CSF analysis was also normal as expressed by normal white cell count, protein and glucose levels. The red blood cells present in some of the samples were the result of traumatic lumbar puncture (LP). Samples were also checked for T-tau, P-tau and Aβ42 levels as previously described [24], and were within normal limits (Figure 1). At the 2nd stage, a total of 49 samples were collected prospectively by recruiting (a) a new group of healthy controls (n=15, collected between August 2012 and January 2013, under the same conditions), who fulfilled the above mentioned criteria (CTRL batch 2) and (b) the patient groups consecutively recruited (between 2010 and 2012) without selection, through a routine process. Patients were divided in two well characterized groups: (i) The DLB group comprised of 16 patients fulfilling the criteria of the third report of the DLB Consortium [25]. Additionally, in order to ensure correct classification, CSF biomarkers - namely total tau (τΤ), amyloid Aβ42 and phospho-tau (τΤ-181) were measured, revealing a non-AD profile. (ii) The AD group comprised of 18 patients with probable Alzheimer’s disease according to the NIA-AA criteria for probable AD, with biomarker support [5]. CSF biomarker levels for both patients and controls are shown in the Table S1.

All patients underwent a detailed evaluation (medical history, physical and neurological examination, blood tests to exclude metabolic causes of dementia, MRI and neuropsychological assessment). Laboratory evaluation included complete blood count, serum electrolytes, blood urea nitrogen, creatinine, glucose, vitamin B12 and thyroid function tests; all results were within normal limits.

### 2: Sample collection

Lumbar puncture was performed at the L4-S1 interspace, between 9–12 AM after overnight fasting. Samples were obtained in polypropylene tubes. Approximately 12 ml of CSF were drawn from each subject, in three fractions of 2, 5 and 5 ml. The first 2 ml-tube was used for routine diagnostic purposes. The following two tubes were immediately (within 30 min) centrifuged at 500xg for the removal of cells, aliquoted (0.75 ml) into several 1 ml polypropylene tubes and stored at –80°C until analysis. They were thawed only once, just before the assay. To avoid any potential variations arising from a rostro-caudal gradient, the same fractions of both patient and control group (2nd tube) were assessed. Samples with more than 50 RBCs in the first tube were rejected from the 2nd stage of the study (see below).

### 3: Determination of α-Syn concentration

The in-house ELISA for the accurate quantification of α-Syn concentration in biological fluids was developed by using two commercially available α-Syn-specific antibodies: the monoclonal Syn-1 (BD Transductions) as capture antibody and the polyclonal C-20 (Santa Cruz) as detection antibody, which was used after its covalent conjugation with HRP [26]. Briefly, each ELISA plate (Corning Costar) was coated for 24 hrs at room temperature with 0.5 μg/ml of Syn-1 antibody (50 μl per well) in 100 mM NaHCO3, pH 9.3. The plates were washed three times in wash buffer (50 mMTris-HCl, 150 mM NaCl and 0.04% Tween-20) and recombinant human α-Syn (Chemicon) (as standard) appropriately diluted in TBST/BSA (10 mM Tris-Cl, pH 7.6, 100 mM NaCl, 0.1% Tween-20 and 1% BSA) or 45 μl of CSF sample mixed with 5 μl 10x TBST was added. To allow antigen binding, plates were incubated at 37°C for 2.5 hrs. After washing three times with wash buffer, 50 μl of HRP-conjugated C-20 antibody (4000x diluted in TBST/BSA) were added to each well and further incubated for 1 hr at ambient temperature. The wells were washed and 50 μl of chemiluminogenic HRP substrate (UptiLight HS ELISA HRP substrate, Interchim) were added to each well. Following incubation for 10 min at ambient temperature, chemiluminescence was integrated for 1 s. Standards and CSF samples were measured in triplicate.

### 4: Haemoglobin test

 To avoid misinterpretation of our results, we wanted to verify that CSF samples showing high levels of α-Syn but < 50 RBCs were not contaminated with RBCs lysed during sample collection and processing. For this reason, haemoglobin levels were determined using the Human haemoglobin ELISA Quantitation Kit (Bethyl Lab Inc) in two CSF samples from each CTRL group (Batch 1 and Batch 2) and three CSF samples from each disease group (DLB and AD) that showed the higher α-Syn levels. All samples analysed had heamoglobin levels <300 ng/ml. The assay was performed according to the manufacturer’s instructions.

### 5: Statistical analysis

All numerical variables were checked for their distribution and for homogeneity of variances by the Shapiro-Wilk’s and Levene’s tests respectively. The levels of α-Syn did not follow the normal distribution, while their variances were heterogeneous. Logarithmic transformation restored the above violations, permitting the use of two-way analysis of covariance (two-way ANCOVA) for comparison among groups. Sex and diagnostic group were introduced as co-factors and age and MMSE as covariates. One-way ANCOVA and Kruskal-Wallis test were also performed when appropriate.

Correlation of α-Syn levels with clinical parameters or various parameters of the CSF (including RBCs) was performed by the parametric Pearson (R_P_) or the Spearman rank correlation (R_S_) coefficients as appropriate.

The diagnostic value of α-Syn was analyzed by Receiver Operating Characteristic (ROC) curve analysis. Analysis of ROC curves, including cut-off values with the optimal combination of sensitivity and specificity, was performed by Medcalc® version 6.16 (MedCalc Software, Mariakerke, Belgium, 2002). Asymptotic *P* values for comparison of areas under the ROC curve with the theoretical area of 0.5 were calculated by SPSS® version 16.0 (SPSS Inc, Chicago, IL, 2007).

## Results

Demographic, clinical and biochemical data of patients and controls are summarized in Table 1.

**Table 1:** Clinical and biochemical data of the studied groups

	CTRL	CTRL	CTRL combined	DLB	AD
	Batch 1	Batch 2	(<50 RBCs)		
N (m / f)	19 (10 / 9)	15 (6 / 9)	22 (11 / 11)	16 (12 / 4)	18 (8 / 10)
Age (y)	70.3 ± 10.3	69.7 ± 8.1	70.6 ± 7.5	72.7 ± 5.8	71.3 ± 8.6
Duration (y)				3.4 ± 3.7	2.9 ± 1.7
MMSE	29 (27 - 30)	29 (27 - 30)	29 (27 - 30)	17 (1 - 21)	17.5 (13 - 27)
WBCs /μl	2 (1 - 6)	0 (0 – 5)	0 (0 – 5)	0 (0 – 4)	0 (0 – 3)
RBCs /μl	75 (0 - 9500)	0 (0 – 40)	0 (0 – 40)	0 (0 – 35)	0 (0 – 40)
Protein mg/dl	25 (9 - 75)	38 (10 - 82)	37.5 (10 - 82)	41 (20 - 72)	35.5 (18 - 55)
α-Syn (pg/ml)	136 (104 - 185)	89 (76 - 109)	93 (76 - 111)	135 (94.5 - 284.8)	99.5 (66.5 - 120.5)
	166.1 ± 85.2	105.9 ± 46.6	106.1 ± 43.3	174.7 ± 103.5	92.1 ± 42.4

Data are shown as mean ± SD or median values (range)

### 1: Optimization and analytical characteristics of a-Syn ELISA

**Figure 2:**
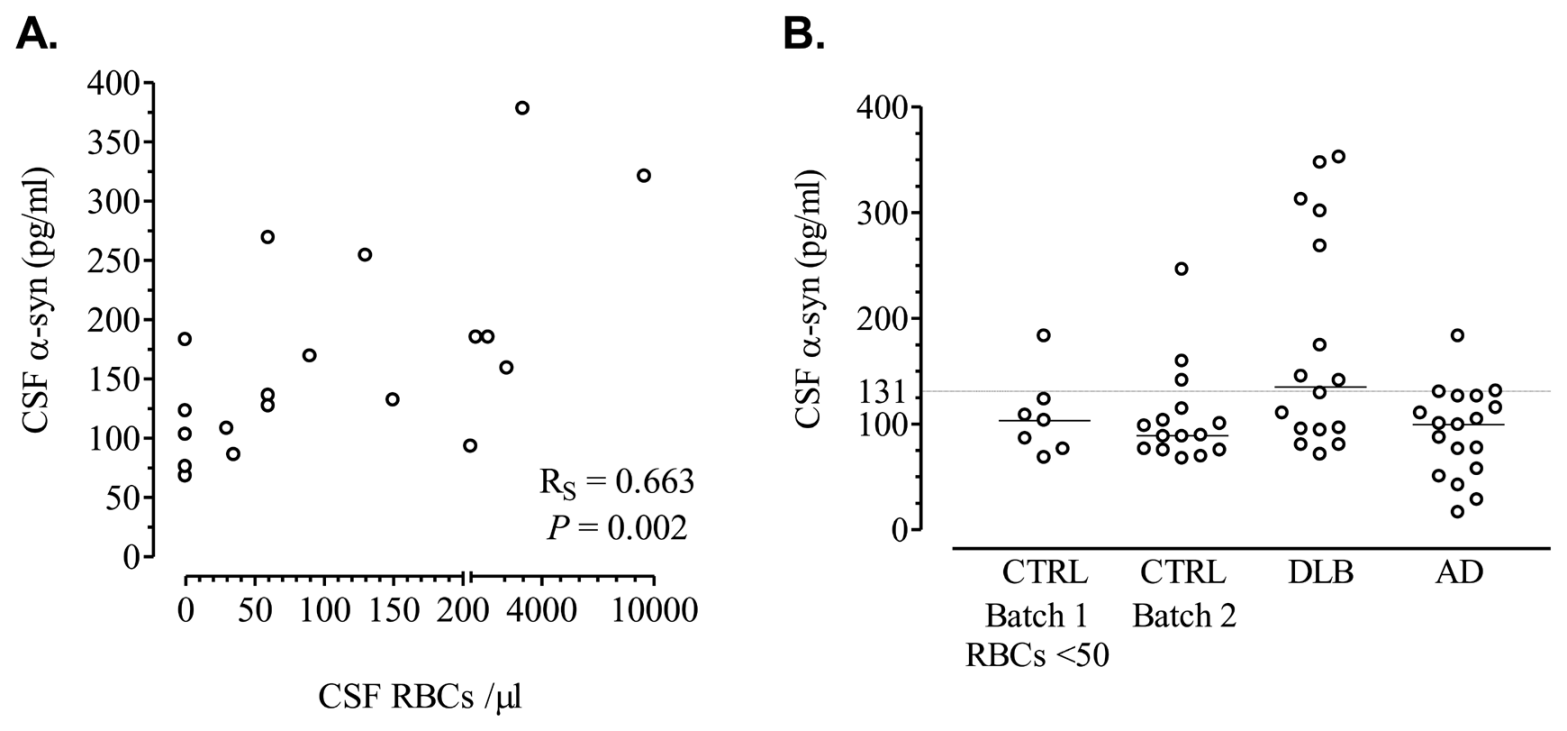
Scatterplots of α-Syn levels. (A) Correlation between α-Syn levels and RBCs in CSF samples of CTRL Batch 1. (B) Scatterplots of CSF α-Syn levels in patients and controls.

**Figure 3:**
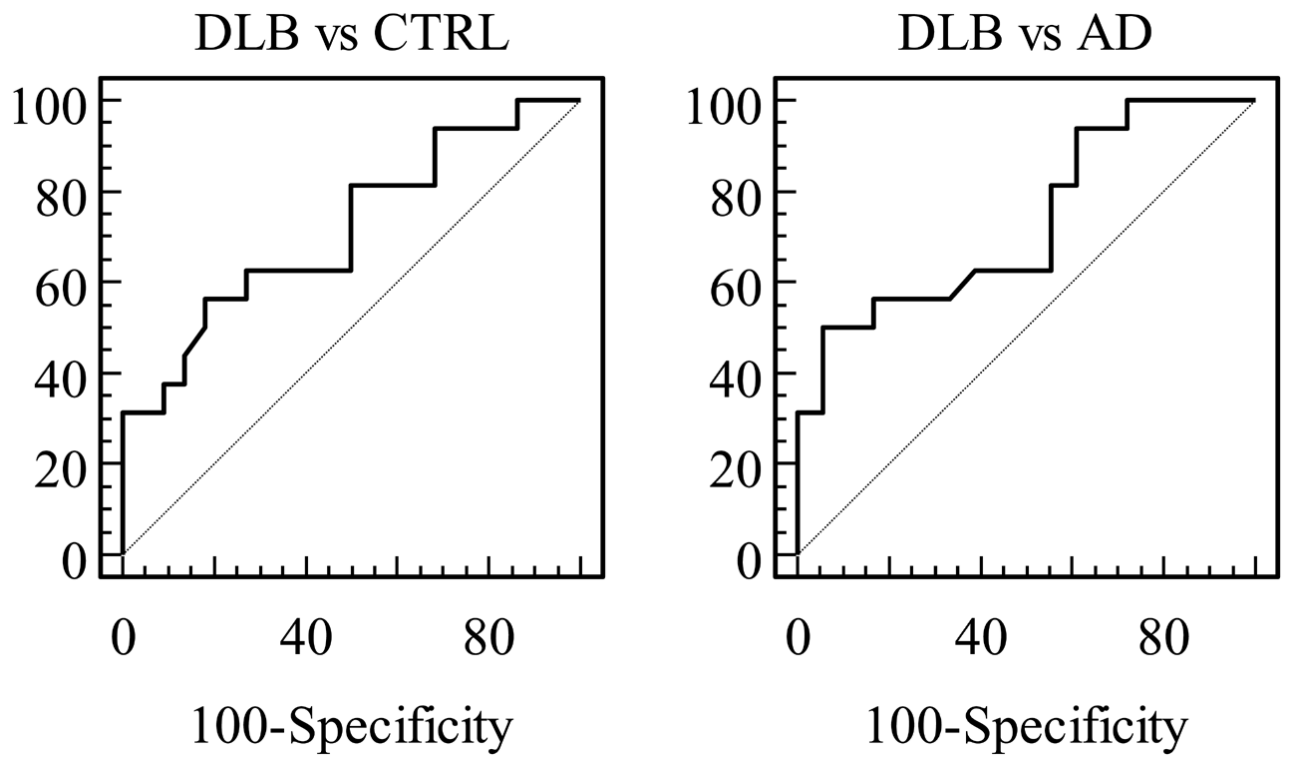
Receiver operating characteristic (ROC) curves of CSF α-Syn for the discrimination between DLB and either CTRL or AD.

The optimal concentration of the capture antibody was determined by measuring the signal over background ratio (S/B) obtained for a low concentration of recombinant α-Syn (0.1 ng/ml) (Figure 2A). Replacement of the Syn-1 by the monoclonal 211 (Millipore) resulted in much lower sensitivity whereas use of 5C2 antibody (Acris Antibodies Inc) failed to detect α-Syn as depicted by the S/B ratios obtained for the measurement of 0.1 ng/ml recombinant α-Syn (Figure 2B). The method was also optimized for the time required for optimal capture of α-Syn. Recombinant α-Syn (0.3 ng/ml) was measured by ELISA following 1.5, 2 or 2.5 hours-incubation at 37°C and the S/B ratios were estimated (Figure 2C). Finally, SSC, PBS and TBS assay buffers supplemented with 0.1% Tween-20 were tested by comparing the S/B ratios obtained for the measurement of 0.1 ng/ml recombinant α-Syn (Figure 2D). For the measurement of human CSF samples, the standard curve should be performed in artificial CSF. However, we have finally used TBST/BSA buffer in our standard curves since we have found that it behaves identically with artificial CSF (Figure 3).

The analytical characteristics of the α-Syn ELISA were determined using the established optimized protocol. The limit of quantitation (LOQ) of the assay was established by analyzing serial dilutions of recombinant human α-Syn (Figure 1A). As demonstrated by the standard curve, the ELISA was highly sensitive detecting as low as 5 pg/ml of recombinant human α-Syn with a S/B ratio of 2. The linear range of the assay extends up to 25 ng/ml. To determine the specificity of the new ELISA, we generated a standard curve using serial dilutions of either recombinant α-Syn or β-Syn. As depicted in Figure 1B, no signal was obtained for the detection of β-Syn even in higher concentrations (Figure 1B).To further assess the specificity of the method, we used the ELISA to quantify α-Syn concentration in cortex homogenates from either α-Syn knock-out (KO), wild-type (WT) or α-Syn transgenic (Tg) mice. Measurement of brain extracts from α-Syn KO mice resulted in non-detectable levels whereas α-Syn was readily detected in WT and Tg mouse brain (Figure 1C). Specifically, α-Syn levels were 3.7 fold higher in the brain extracts from Tg mice compared with WT mice (109.4 ± 3.8 vs 29.3 ± 0.2 µg/ml for the Tg and WT mice, respectively, mean ± SD, n = 4). These results are in accordance with previous measurements where α-Syn levels in the brain extracts of WT and Tg mice were estimated using western blotting and densitometry. 

The intra-assay variation for 0.1-25 ng/ml of recombinant α-Syn ranged from 1 to 7%. The day-to-day reproducibility of the assay was assessed by performing similar calibration graphs during a period of 6 months (n=7 curves). The average of the S/B ratios for each concentration were estimated and plotted versus the input of recombinant α-Syn, where the error bars correspond to the reproducibility of the assay for that period (Figure 1D). In addition, we used different calibration graphs obtained over the period of 6 months to measure 0.3 ng/ml of recombinant α-Syn (input). The results showed a day-to-day reproducibility of 9.7% (mean ± SD= 0.31 ± 0.03 ng/ml, n = 8).

### 2: Correlation of a-Syn concentration with RBCs

#### 2.1: CTRL Batch 1 (6y storage, RBCs range 0-9500/μl)

A positive correlation was noted between α-Syn levels and RBC content of the CSF (Figure 2A). This positive correlation was evident at all levels of CSF contamination by blood, except for those samples with less than 50 RBCs/μl (Table 2). Then, control samples were stratified according to their RBC content in 3 subgroups: <50 RBCs (n = 7), 50–200 RBCs (n = 6) and >200 RBCs (n = 6). Median (quartiles) values of α-Syn were 103 (76–123), 152 (127–211.5) and 185 (93–253) pg/ml respectively and the difference was statistically significant (ANOVA after logarithmic transformation, *P* = 0.017). A post-hoc test for trend showed a statistical significant increase of α-Syn levels among the 3 subgroups with increasing RBC content (R^2^ = 0.349, *P* = 0.008), while Newman-Keuls post-hoc tests revealed that both the 50–200 and the >200 RBCs subgroups had higher α-Syn levels as compared to the <50 RBCs subgroup (*P* = 0.029 and 0.02, respectively).

**Table 2:** Correlation between α-Syn levels and RBC content of the CSF in CTRL batch 1.

CSF RBCs	R_S_	*P* value
Entire group	0.663	0.002
<2000	0.541	0.031
<200	0.597	0.031
<100	0.549	0.08
<50	–0.04	0.92

No correlation was noted between α-Syn and white blood cells (R_S_ = 0.04, *P* = 0.88), CSF protein (R_S_ = –0.06 *P* = 0.79) and MMSE (R_S_ = 0.04, *P* = 0.87). A positive correlation with age did not reach statistical significance (R_P_ = 0.33, *P* = 0.15). No significant difference was observed between males and females (*P* = 0.36).

#### 2.2: CTRL Batch 2 (6mo storage, RBCs <50/μl)

No correlation was noted between α-Syn levels and RBCs (R_S_ = 0.001, *P* = 0.9), WBCs (R_S_ = –0.002, *P* = 0.97) and MMSE (R_S_ = 0.06, *P* = 0.81), while no difference was observed between males and females (*P* = 0.85). Positive correlations with age (R_P_ = 0.4, *P* = 0.13) and CSF protein (R_S_ = 0.29, *P* = 0.28) did not reach statistical significance.

The levels of α-Syn in this second batch (see Table 1) were compared with the 3 subgroups of the CTRL batch 1 (2-way ANCOVA, *P* = 0.0029, with no significant effect of sex, sex by group, age and MMSE). The controls of batch 2 had lower levels as compared to the subgroups of batch 1 with 50-200 and >200 RBCs (Newman-Keuls post hoc tests *P* = 0.02 and 0.004 respectively). However, they did not differ significantly as compared to the subgroup of batch 1 having < 50 RBCs in CSF (*P* = 0.67).

### 3: Comparison with patient groups

Since α-Syn levels in CTRL batch 2 were almost identical with the subgroup of CTRL batch 1 with <50 RBCs/μl, both were combined in a common CTRL group and compared to the levels of patients with AD and DLB (Table 1, Figure 2B). Finally, data from 22 healthy controls (CTRL), 16 DLB and 18 AD patients were used in the statistical evaluation.

Diagnostic group (but not sex or sex by group) showed a significant effect (*P* = 0.0029). Age and MMSE did not affect the model significantly. Post hoc Neuman-Keuls tests revealed a significant increase of α-Syn levels in DLB patients as compared to the CTRL and AD groups (*P* = 0.049 and 0.01 respectively), while the AD group did not differ as compared to CTRL. 

### 4: ROC analysis

Levels of α-Syn showed a statistically significant discrimination between DLB and either CTRL or AD (Figure 3, Table 3). 

**Table 3:** Receiver operating characteristics (ROC) curve analysis for α-Syn.

Cut-off value	Area under the curve	Sensitivity (%)	Specificity (%)	*P* value*
DLBvsCTRL 123 pg/ml	0.71 (0.55–0.85)	56.2 (29.9–80.2)	81.8 (59.7–94.7)	0.026
DLB vs AD 131 pg/ml	0.73 (0.55–0.87)	50.0 (24.7–75.3)	94.4 (72.6–99.1)	0.022

Values in parentheses indicate 95% confidence intervals as computed by MedCalc 6.16. *Asymptotic *P* values as computed by SPSS 16.0 for comparison with diagonal line (area under the ROC curve 0.5)

## Discussion

Quantitative analysis of CSF α-Syn levels with ELISA presents great variation among studies. The variation ranges from a few hundreds of pg/ml to several ng/ml in both the control group, as well as in different patient groups. This wide variation (100-fold difference) probably reflects technical/methodological differences and/or different species of α-Syn measured, rather than an actual biological cause. In the majority of the studies, the control group comprised of patients suffering from other neurological diseases and only a few studies [19,21,27,28] have included a healthy control group. Other studies included no control group [11,16].

Among various confounders of CSF α-Syn quantification, blood contamination has been widely recognized, as α-Syn is highly abundant in the blood. Especially in erythrocytes, its levels are markedly higher than those in plasma and/or serum [29]. However, blood contamination has not been taken into consideration in several studies. Importantly, in studies where a blood control has been included, the cutoff value used was 500 RBCs/μl, a number 10-fold higher than the value (50 RBCs/µl) suggested by the recent recommendations [22]. Only Aerts et al used the cut-off of <50 RBCs in their study [20]. Others have estimated the levels of hemoglobin in their samples as a measure of blood contamination, with cutoff values ranging from <200 ng/ml to <1000ng/l [21,27].

In the present study, we have shown a very strong correlation of α-Syn levels with RBCs, which disappeared only when <50 RBCs/μl were present in the first collection tube. This suggests that in the second cell-free collection tube almost no RBCs were present, and contamination, if any, might have resulted by a few RBCs undergone hemolysis before centrifugation. We have also tried to eliminate other confounders which could influence the levels of α-Syn. Diurnal variation was avoided by performing lumbar puncture in the morning. Food intake was controlled, since samples were collected following overnight fasting. We have also avoided the appearance of a concentration gradient between samples by measuring the same CSF fraction. Other processing details included use of polypropylene tubes to minimize possible tube-wall absorbance, filling of the tube by the same amount of CSF (3/4 in 1ml tubes) and immediate sample processing. Regarding the length of storage, α-Syn in CSF seems to be stable when samples are treated and stored under optimum conditions, since no differences in α-Syn levels were observed between old (6 years storage) vs. new (6 months) CTRL samples. This is the first study contacted with such strict adherence to the very recent recommendations [22].

The measurements presented in this study have been performed using a new ELISA with high sensitivity and selectivity. Importantly, the antibodies utilized by this method (Syn1 and C-20) are both commercially available, with well-characterized, known epitopes, which ensure recognition of the α-Syn monomer. However, due to the topology of the epitopes, we have verified that α-Syn detected by this assay corresponds to the total levels of the protein (oligomeric and monomeric). Other monoclonal antibodies, such as 211 and 5C2, have been tested as capture antibodies in our assay format using C-20 as detection antibody. From these antibodies, 211 showed a preferential detection of oligomeric species probably due to the epitopes of the two antibodies being in close proximity, if not overlapping with each other. On the other hand, 5C2 antibody displayed almost no binding with the target protein. This lack of binding could either be due to limited accessibility of 5C2 epitope (which is located in the NAC domain of α-Syn) or to steric hindrance between 5C2 and C-20 antibodies. These findings highlight the importance of selecting the appropriate antibody for the development of a new ELISA assay in a way to allow detection of specific species or quantification of the total amount of target protein.

No significant effect of age, sex, and MMSE on α-Syn was observed in CTRL or in the patient groups. Other authors have reported a decrease with age [10,16,18] while van Geel et al (2008) [30] found no change as opposed to Hong et al (2010) [27], who report an increase especially in normal controls. The above discrepancies could be attributed mainly to methodological pre-analytical differences and possibly to the range of age in the studied groups, while differences in the ELISA method used and/or ethnic differences cannot be excluded.

The main finding of the present study is the significant increase of α-Syn levels in DLB as compared to the CTRL and AD groups, while AD did not differ as compared to the CTRL group. This is the first study to our knowledge that reports an increase in CSF total α-Syn concentration as detected by our optimized ELISA. Previous studies, have reported either decreased [10-15] or unchanged [16-21] total α-Syn levels. These studies are not comparable, since they differ in both pre-analytical and analytical factors mentioned above. Even among studies that have contacted strict blood contamination control [21,27] and have used similar method (Luminex), Hall et al reported increased CSF α-Syn levels in AD vs. controls, while Hong et al found no differences for the same population. It is important to note that the Luminex-based assays used, employed different α-Syn antibodies and therefore could detect different α-Syn species. Interestingly, the same combination of antibodies used in different assays (Luminex vs ELISA) has given opposite results in similar patient cohorts [18,21].

According to the published criteria for biological markers of the Reagan Research Institute of Alzheimer’s Association and the National Institute on Aging (The Working Group on Molecular and Biochemical markers of AD) [31] in order to be clinically useful, a diagnostic marker should have sensitivity, specificity and positive predictive value approaching or exceeding 80-85%. In the present study, ROC analysis for the discrimination of DLB vs. either CTRL or AD revealed a very good specificity (at the range of 82-90%); however, sensitivity was modest (50-56%). Clinical-pathologic correlations applying current diagnostic criteria for probable DLB present high variability in reported sensitivity and specificity rates (0.22–0.83 and 0.79–1.00 respectively), with the majority of misdiagnoses to be patients with AD [32]. This is probably reflected in clinical studies as well. Decreased striatal binding of radioactive tracers, as indicated by abnormal dopamine transporter activity on SPECT or PET imaging, has been shown to have good sensitivity and specificity to DLB [33], and is considered the most reliable biological diagnostic marker for this condition. However, there are clinical and pathologic studies [34,35] which indicate that such abnormal scans may also be observed in some patients with AD. Thus, alternative or additional methods, such as CSF biomarkers reflecting the pathophysiological process of the disease are required to increase diagnostic confidence.

It has been shown that under normal conditions full length α-Syn is detected in CSF [7,26]. Investigation of its origin in CSF has shown that despite its higher levels in peripheral blood products, neurons of the brain and the spinal cord represent the principal source of α-Syn in human CSF [8]. Diurnal or sinusoidal fluctuations are not present over 33h [17] either in healthy elderly controls or in patients with AD, while no significant rostro-caudal gradient concentration has been reported [8,27]. Under pathological conditions, abnormal release of α-Syn in the extracellular space might be the result of intracellular aggregation or neuron damage, alterations in SNCA gene transcription, mRNA splicing or protein processing, reduced CSF flow with reduced clearance rate from CSF, or other yet unidentified factors. The effect of dopaminergic or other medication still has to be elucidated.

An inherent limitation in the majority of biomarker studies (the present study included) is the lack of pathological verification. Although our clinical sample is rather small, the main strength of our study is the implementation of standardized operating procedures and the use of a healthy control group for comparisons. Additionally, in order to minimise incorrect classification of patients as much as possible, extensive investigation, implementation of the latest clinical criteria, enhanced by determination of the already established CSF biomarkers, plus a short follow up period were applied. The importance of confirmation of these results in pathologically validated series in future studies is required.

In conclusion, the results of the present study are compatible with an increase of CSF total α-Syn in DLB as compared to both CTRL and AD groups. Although specificity was high, sensitivity did not exceed 60%. However, this is the first study presenting the possible diagnostic value of CSF α-Syn in terms of sensitivity and specificity, thus rendering CSF α-Syn as a potential diagnostic tool for DLB. Further studies are required as to whether combination of α-Syn with other biomarkers may increase the diagnostic value not only from CTRL and AD, but from other synucleinopathies as well.

## Supporting Information

Figure S1 **Scatterplots of CSF levels of total tau (τT), amyloid Aβ42 and phospho-tau (τP-181) in the studied groups.** Horizontal bars indicate median values and horizontal lines indicate cut-off values of our laboratory (300 pg/ml, 490 pg/ml and 58 pg/ml, respectively).
(TIF)

Figure S2 **Optimization studies for the α-Syn ELISA.** (A) The optimal concentration for the capture antibody (0.5 µg/ml) determined by the measurement of 0.1 ng/ml recombinant α-Syn. (B) Use of 211 monoclonal antibody results in a 4-fold decrease in assay sensitivity. Capture antibodies were used at 0.5 µg/ml and the concentration of recombinant α-Syn measured was 0.1 ng/ml. (C) Determination of the time required for the optimal binding of α-Syn (0.3 ng/ml) to the capture antibody. (D) The assay buffer (TBST) was selected by the measurement of 0.1 ng/ml recombinant α-Syn.
(TIF)

Figure S3 **aCSF behaves identically with TBST in our assay format.** Recombinant α-Syn (0.1, 0.3, 0.9 ng/ml) was diluted either in TBST or aCSF and the calibration curves were presented in parallel.
(TIF)

Table S1 **CSF levels of total tau (τ_T_), amyloid Aβ42 and phospho-tau (τ_P-181_) in the studied groups.**
(DOC)
