# Supplementary figures and images for: The Diagnostic Value of CSF α-Synuclein in the Differential Diagnosis of Dementia with Lewy Bodies vs. Normal Subjects and Patients with Alzheimer’s Disease
Source: PLoS One. 2013 Nov 25;8(11):e81654. doi: 10.1371/journal.pone.0081654 (PMC3840054; doi:10.1371/journal.pone.0081654)

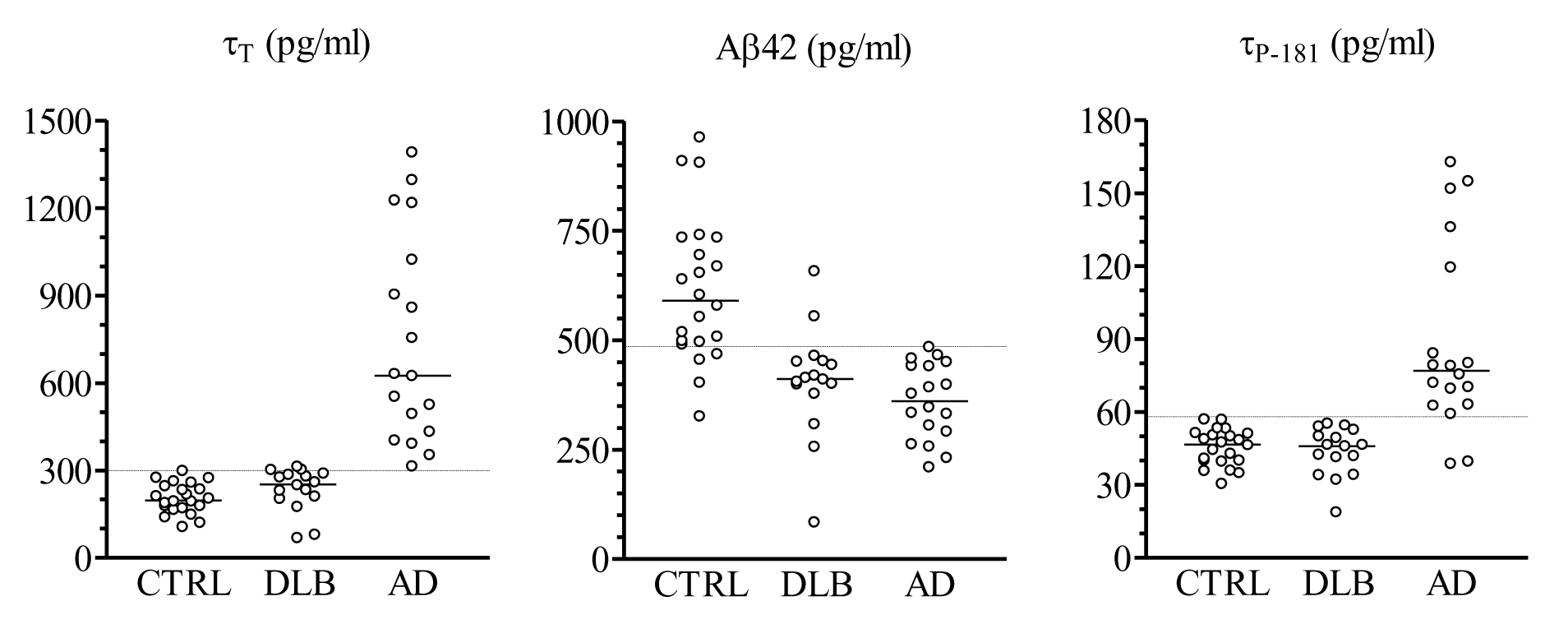

Supplement: Figure S1 — Scatterplots of CSF levels of total tau (τT), amyloid Aβ42 and phospho-tau (τP-181) in the studied groups. Horizontal bars indicate median values and horizontal lines indicate cut-off values of our laboratory (300 pg/ml, 490 pg/ml and 58 pg/ml, respectively). (TIF) [file pone.0081654.s001.tif]

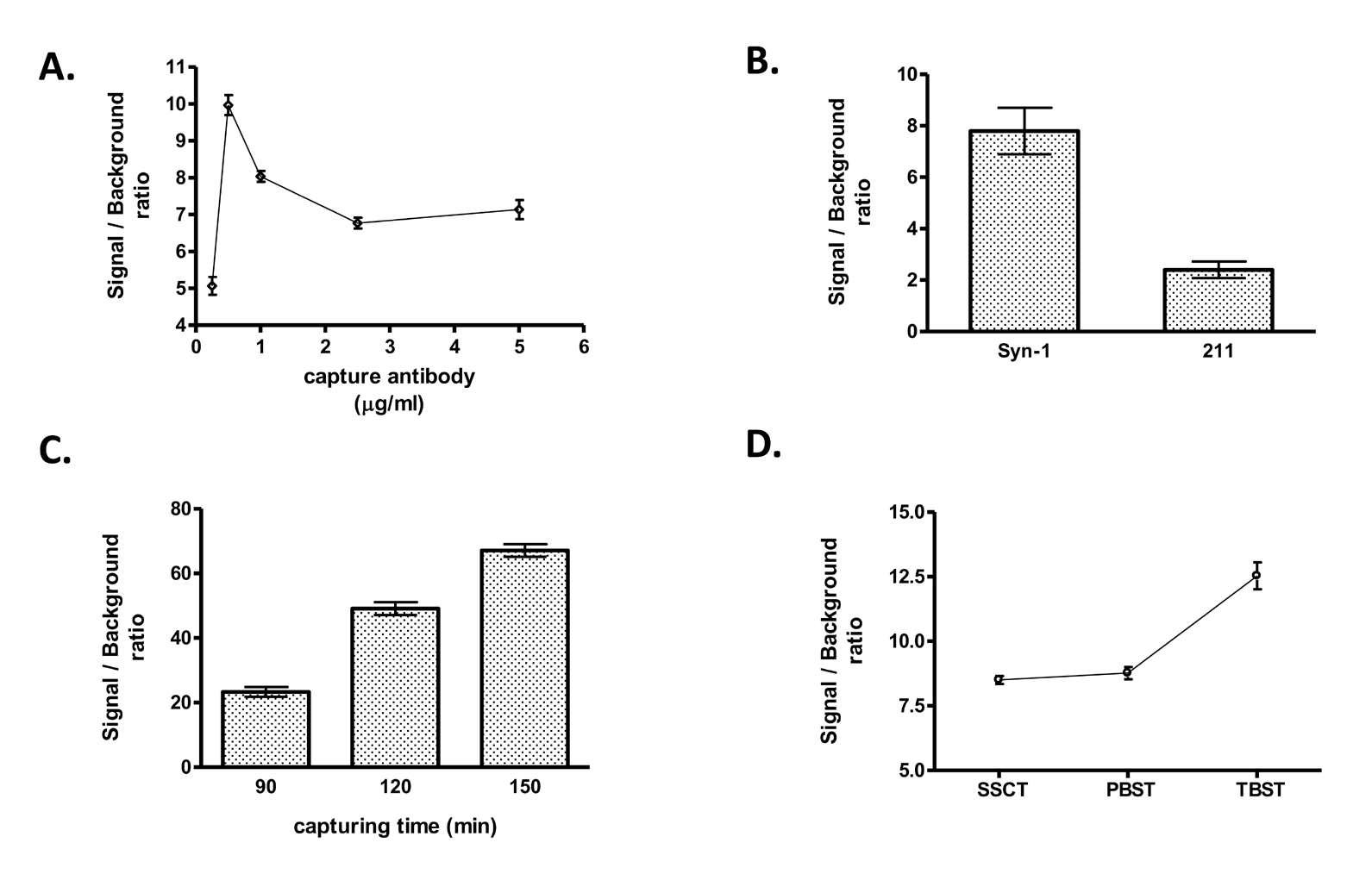

Supplement: Figure S2 — Optimization studies for the α-Syn ELISA. (A) The optimal concentration for the capture antibody (0.5 µg/ml) determined by the measurement of 0.1 ng/ml recombinant α-Syn. (B) Use of 211 monoclonal antibody results in a 4-fold decrease in assay sensitivity. Capture antibodies were used at 0.5 µg/ml and the concentration of recombinant α-Syn measured was 0.1 ng/ml. (C) Determination of the time required for the optimal binding of α-Syn (0.3 ng/ml) to the capture antibody. (D) The assay buffer (TBST) was selected by the measurement of 0.1 ng/ml recombinant α-Syn. (TIF) [file pone.0081654.s002.tif]

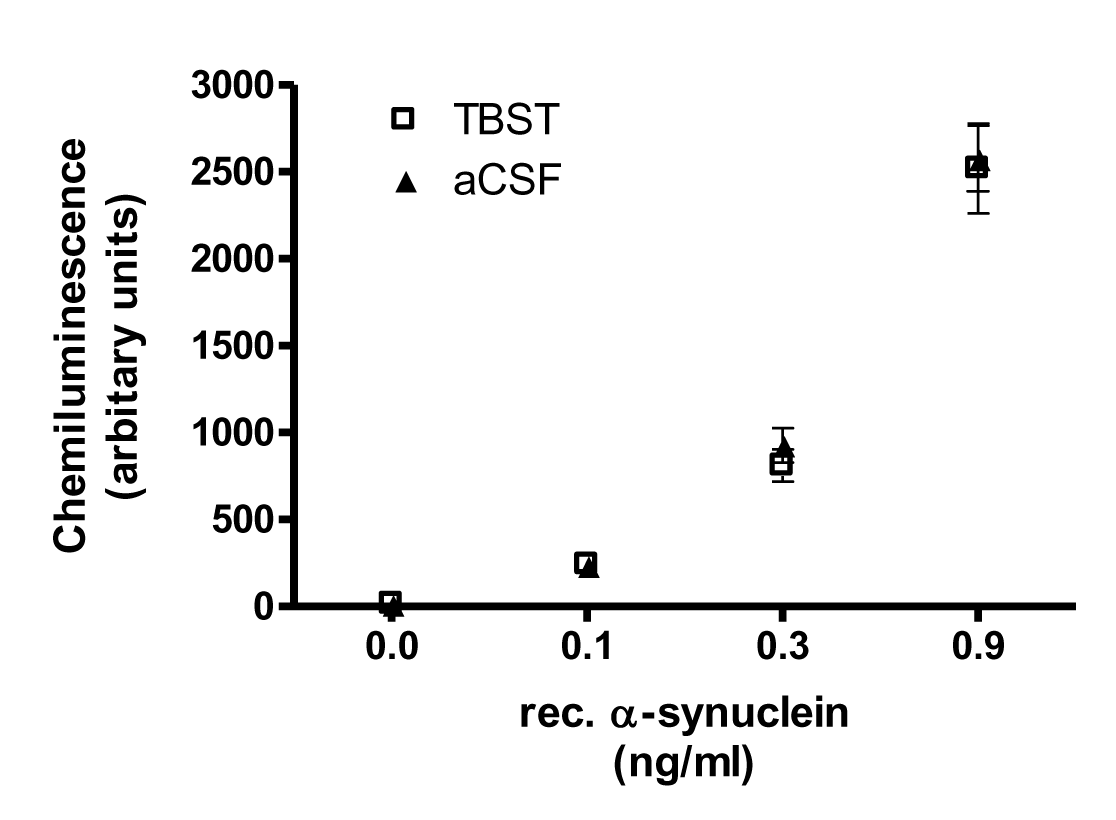

Supplement: Figure S3 — aCSF behaves identically with TBST in our assay format. Recombinant α-Syn (0.1, 0.3, 0.9 ng/ml) was diluted either in TBST or aCSF and the calibration curves were presented in parallel. (TIF) [file pone.0081654.s003.tif]
