# Supplementary material for: The Diagnostic Value of CSF α-Synuclein in the Differential Diagnosis of Dementia with Lewy Bodies vs. Normal Subjects and Patients with Alzheimer’s Disease
Source: PLoS One. 2013 Nov 25;8(11):e81654. doi: 10.1371/journal.pone.0081654 (PMC3840054; doi:10.1371/journal.pone.0081654)
Supplement: Table S1 — CSF levels of total tau (τT), amyloid Aβ42 and phospho-tau (τP-181) in the studied groups. (DOC) [file pone.0081654.s004.doc]

| **Supplemental Table.** CSF levels of total tau (τ_T_), amyloid Aβ42 and phospho-tau (τ_P-181_) in the studied groups. Biomarkers were measured by Double sandwich, enzyme-linked immunosorbent assay (ELISA) commercial kits (“Innotest hTau antigen”, “Aβ42” and “phospho-tau_181_”, Innogenetics, Belgium) | | | | |
| --- | --- | --- | --- | --- |
|  |  |  |  |  |
|  |  |  |  |  |
|  | CTRL  (combined < 50RBCs) | DLB | AD | *P* values* |
|  |  |  |  |  |
|  |  |  |  |  |
| τ_T_ (pg/ml) | 197 (172 – 238) | 252 (205 – 285) | 625 (461 – 960) *^b^* | < 0.0001 |
|  | 202.2 ± 52.2 | 232.4 ± 74.0 | 741.6 ± 355.1 |  |
| Aβ42 (pg/ml) | 590 (496 – 713) *^a^* | 411 (387 – 451) | 361 (297 – 440) | < 0.0001 |
|  | 614.3 ± 168.0 | 404.8 ± 124.1 | 358.6 ± 87.2 |  |
| τ_P-181_ (pg/ml) | 46.6 (39.8 – 50.5) | 45.9 (37.5 – 51.1) | 77.0 (66.1 – 101.5) *^b^* | < 0.0001 |
|  | 45.16 ± 7.45 | 43.45 ± 9.92 | 88.51 ± 38.80 |  |
|  |  |  |  |  |
| Most of the variables did not follow the normal distribution and variances were heterogeneous. Thus, data are presented as median values (25th – 75th percentile). However, for compatibility with recently developed biomarker databases they are also presented as mean ± SD.  * Kruskal-Wallis test, followed by Dunn’s post-hoc tests.  *^a^* *P* < 0.001 vs both DLB and AD. *^b^* *P* < 0.001 vs both CTRL and DLB | | | | |
